# P009. A case of new daily persistent headache treated with botulinum toxin type A

**DOI:** 10.1186/1129-2377-16-S1-A119

**Published:** 2015-09-28

**Authors:** Marco Trucco, Luigi Ruiz

**Affiliations:** Department of Neurology, Headache Centre, Santa Corona Hospital, Pietra Ligure, Italy; Department of Neurology, Headache Centre, SS. Antonio e Biagio Hospital, Alessandria, Italy

## Background

New daily persistent headache (NDPH) is a primary headache disorder, characterized by chronic and unremitting daily headache with abrupt onset and more than three months in duration. It lacks typical clinical features, the pain being suggestive of chronic migraine without aura or tension-type headache. It may be self-limiting within months or years without therapy, or be refractory to most treatments.

## Case report

M.L., female, 19 years old, first seen in August 2013, with left renal hypoplasia and Raynaud's phenomenon, referred since December 24, 2012 (this date was specifically remembered) the onset of a daily, continuous and unremitting headache localized bilaterally in the forehead and occipital region, with pressing/tightening features, tension in both shoulder muscles and lack of general and local autonomic symptoms. The intensity of pain was variable, with paroxysms of disabling intensity. No history of familial headache. No psychiatric features were diagnosed. The patient did not remember head or cervical traumas.

Neurological examination was unremarkable. EEG and angio-RM, as well as CSF examination, were negative.

The pain was refractory to various symptomatic and prophylactic drugs, including NSAIDs, acetaminophen, dipyrone, rizatriptan, and amitryptiline, propranolol, flunarizine, pizotifen, pregabalin, duloxetin and to non-pharmacological therapy, such as, acupuncture and positioning of bite-plane. A cycle of i.v. acyclovir was performed without success.

She was treated with botulinum toxin type A 195 U s.c. since January 2014, with cycles every three months. The pain partly relieved after the first cycle and subsided almost completely after the third cycle, becoming tolerable although the patient never became headache free. She is still regularly in treatment, and consistently experienced relief of headache immediately after each cycle, with subsequent worsening of pain, without side effects.

## Discussion

The clinical characteristics of the headache, normality of examinations and refractoriness to most therapies were in accordance with NDPH. Other cases of responsiveness of this headache entity to botulinum toxin have been described[1], but the therapy has still not been approved in Italy for NDPH, due to the lack of controlled studies. In this case we can reasonably exclude a spontaneous remission of the headache because of the persistence of less severe pain, clearly relieved by the therapy. Botulinum toxin type A was the only effective treatment in this case, and strongly contributed to the improvement of the quality of life of the patient.

## Conclusions

We present this case report as a stimulus to perform more observations to test the validity of botulinum toxin in chronic refractory primary headaches, including NDPH.

Written informed consent to publication was obtained from the patient(s).
